# Supplementary material for: Delineating the Role of Aedes aegypti ABC Transporter Gene Family during Mosquito Development and Arboviral Infection via Transcriptome Analyses
Source: Pathogens. 2021 Sep 2;10(9):1127. doi: 10.3390/pathogens10091127 (PMC8470938; doi:10.3390/pathogens10091127)
Supplement: Supplementary file 1 [file pathogens-10-01127-s001.zip › Supplementary files pathogens-1343090/Supplementary Tables pathogens-1343090.pdf]

Supplementary Table S1. List of primers used for qPCR to validate the ABC transporter genes expression during mosquito development and post-arboviral infection (as mentioned in materials and methods Sections 4.5–4.6).

**Gene Name**	**Forward primer**	**Reverse primer**	**Source**
AaeABCC9	CTTCCCAGACGAACAGCCTA	CCTTGACAATGACCGGCAAA	Present study
AaeABCC13	TTGCTCTCGATTACCTGGGG	ACACGATGTCTCCCTTCTGG	
AaeABCE1	AGGAGAACTGCAGCGTTTTG	TCATCCGGCTCCAGATTACC	
AaeABCF2	CCAGTGCGTTATGGAAGTCG	GCAAGCGTCCAAATCGAGAT	
AaeABCG11	TGACGGTGCTGGAGAATATG	CGTAGAGGCCAAGTACAGTTAG	
AaeABCG13	GAAACGATCGCCATGCAAAC	ACGATACAGGTCAGCAGGAG	
AaeActin	GAACACCCAGTCCTGCTGACA	TGCGTCATCTTCTCACGGTTAG	[65]
AaeS6	CGTCGTCAGGAACGTATCCG	TCTTGGCAGCCTTAGCAGC	[56]

Supplementary Table S2. List of immunity related transcription factors identified in the 5' UTR sequences of representative ABC transporters and their functions [67].

Transcription Factor	Functions of Transcription factor
Rel	Innate immune response, Toll signaling pathway
STAT	Defense response, The Janus kinase-STAT pathway is also connected to insect early development
Ets21C	Defense response to bacterium
Bgb	Regulation of hemocyte proliferation
Cnc	Response to oxidative stress
Schlank (Sch)	SREBP signaling pathway, a sterol response element-binding transcription factor (SREBP) to result in up-regulation of target gene transcription
