# Supplementary figures and images for: Delineating the Role of Aedes aegypti ABC Transporter Gene Family during Mosquito Development and Arboviral Infection via Transcriptome Analyses
Source: Pathogens. 2021 Sep 2;10(9):1127. doi: 10.3390/pathogens10091127 (PMC8470938; doi:10.3390/pathogens10091127)

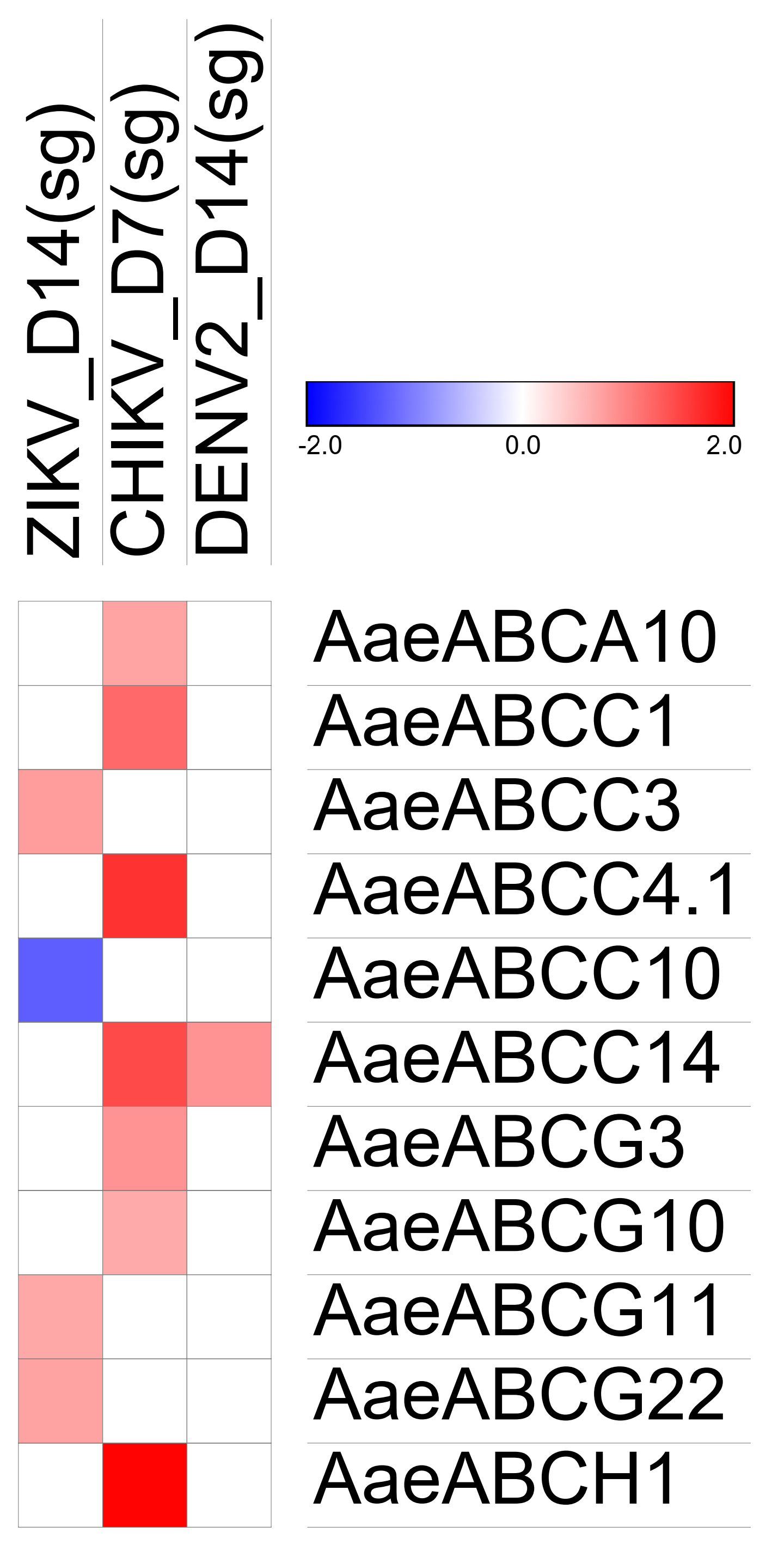

Supplement: Supplementary file 1 [file pathogens-10-01127-s001.zip › Supplementary files pathogens-1343090/Supplementary Figure S2.tif]

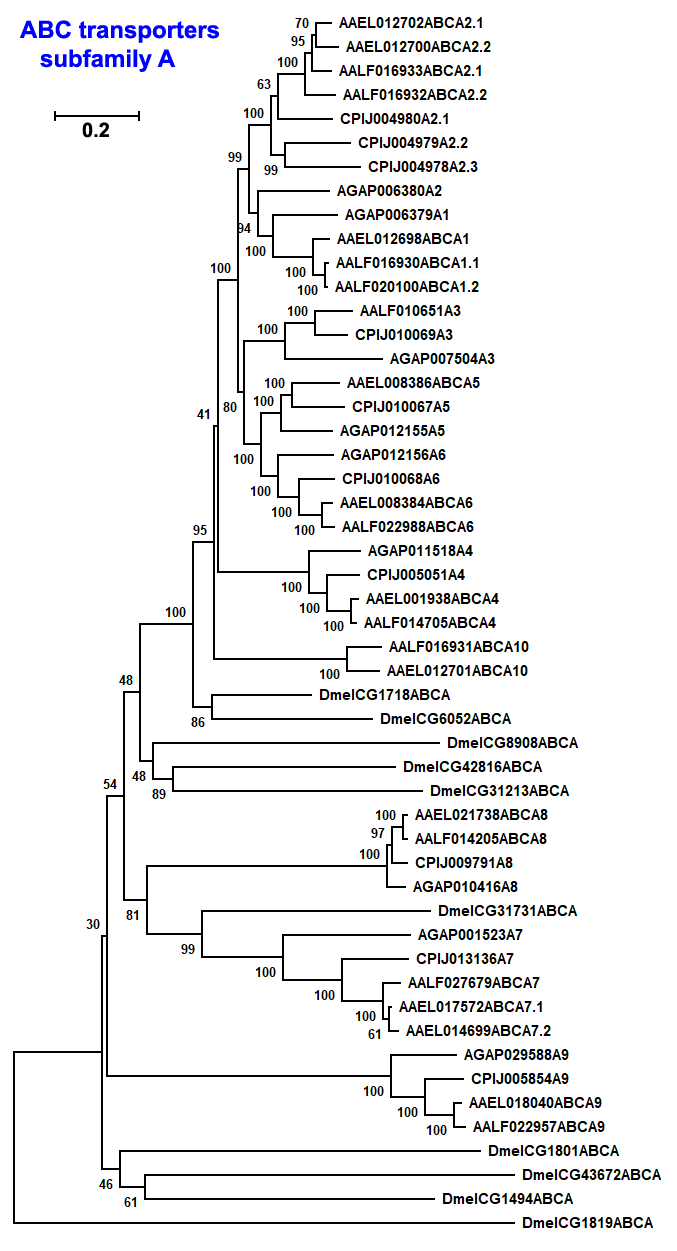

Supplement: Supplementary file 1 [file pathogens-10-01127-s001.zip › Supplementary files pathogens-1343090/Supplementray Figure S1A.tif]

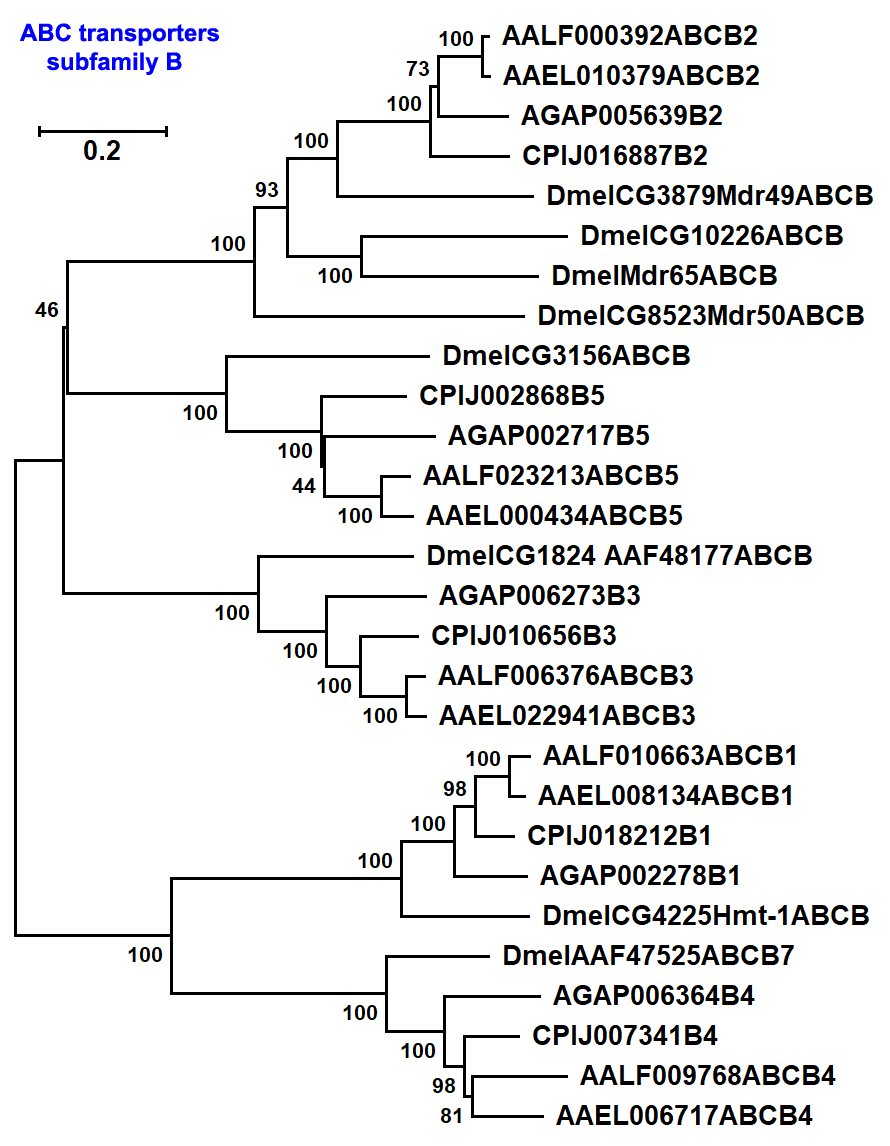

Supplement: Supplementary file 1 [file pathogens-10-01127-s001.zip › Supplementary files pathogens-1343090/Supplementray Figure S1B.tif]

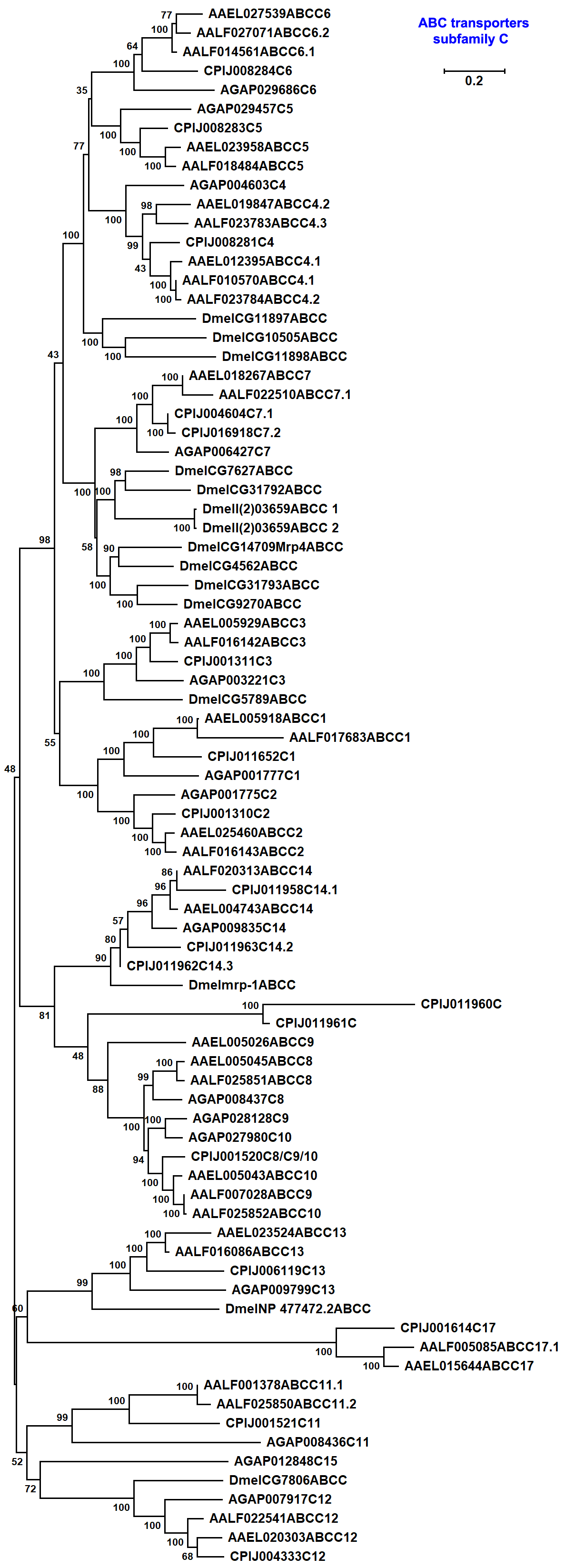

Supplement: Supplementary file 1 [file pathogens-10-01127-s001.zip › Supplementary files pathogens-1343090/Supplementray Figure S1C.tif]

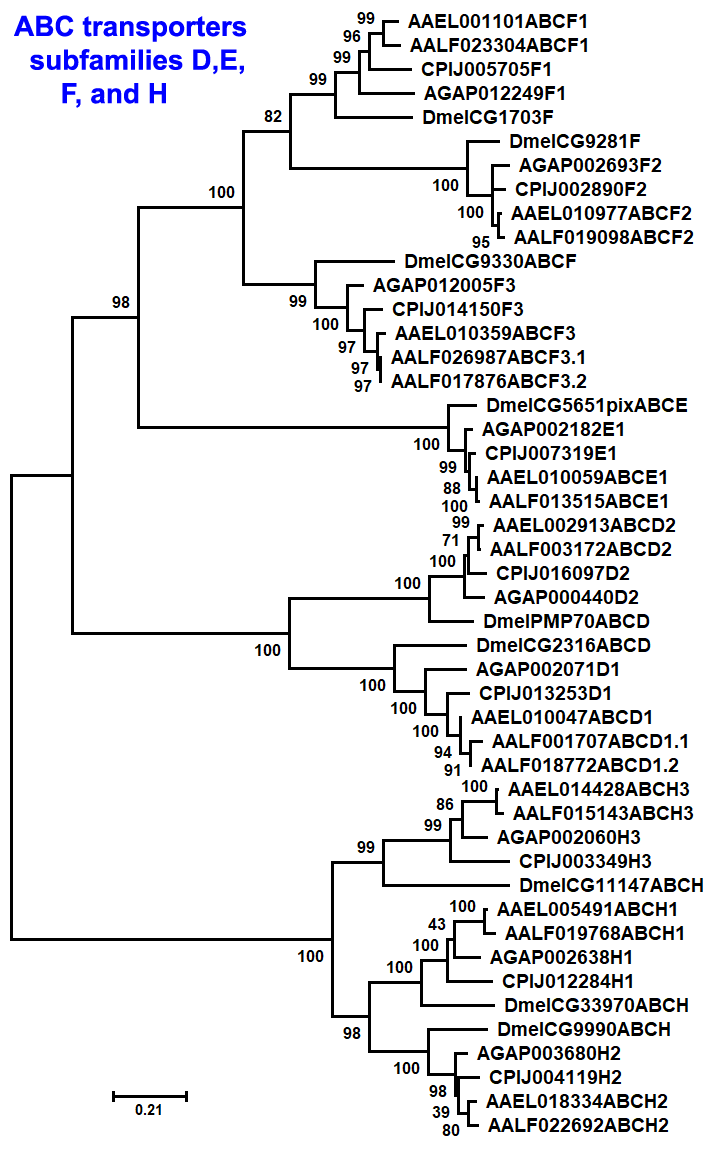

Supplement: Supplementary file 1 [file pathogens-10-01127-s001.zip › Supplementary files pathogens-1343090/Supplementray Figure S1D.tif]

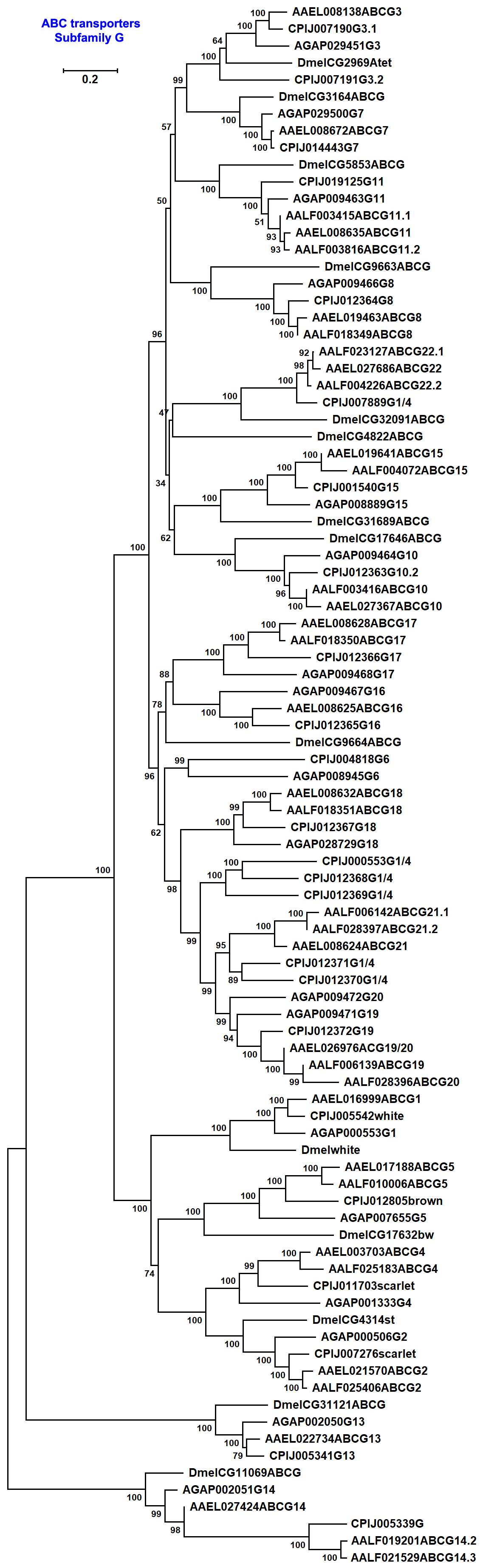

Supplement: Supplementary file 1 [file pathogens-10-01127-s001.zip › Supplementary files pathogens-1343090/Supplementray Figure S1E.tif]
